# Editorial: Methods and applications in inflammation pharmacology

**DOI:** 10.3389/fphar.2022.1108263

**Published:** 2022-12-12

**Authors:** Artur Schmidtchen, Haris Mirza, Mariena J. A. van der Plas, Aftab Nadeem, Manoj Puthia

**Affiliations:** ^1^ Division of Dermatology and Venereology, Department of Clinical Sciences, Lund University, Lund, Sweden; ^2^ Copenhagen Wound Healing Center, Bispebjerg Hospital, Department of Biomedical Sciences, University of Copenhagen, Copenhagen, Denmark; ^3^ Department of Immunobiology, Yale University School of Medicine, New Haven, CT, United States; ^4^ Department of Pharmacy, University of Copenhagen, Copenhagen, Denmark; ^5^ Department of Molecular Biology, Umeå University, Umeå, Sweden

**Keywords:** inflammation, pharmacology, innate immunity, preclinical, animal models

The body has its own ways of defending itself and responding to insults (Delves and Roitt, 2000). Inflammation is one of the most vital biological responses of the immune system. In the body, inflammation can be triggered by various harmful stimuli such as pathogens, toxins, tissue damage, radiation, and autoimmune disorders. The inflammatory process can be acute or chronic in nature and it can affect vital organs and other tissue compartments. Although inflammation is mostly beneficial, excessive inflammation can lead to discomfort, loss of function of organs, and even death (Chen et al., 2018; Furman et al., 2019). The immune system mostly knows how to respond judiciously but sometimes does not know how much to respond, leading to uncontrolled inflammation. During these circumstances of uncontrolled inflammation, pharmacological interventions are needed. Novel interventions, for example, may include the precision medicine (Ashley, 2016), targeted delivery (Srinivasarao and Low, 2017), RNA-based therapies (Guo et al., 2016; Zhu et al., 2022), therapeutic host defense peptides (Mookherjee et al., 2020; Puthia et al., 2020), and innate immune modulation (Kanzler et al., 2007; Puthia et al., 2016). A variety of cell culture models, reporter assays, and animal models including transgenic and humanized mouse models play a key role in studying the molecular mechanisms of inflammation (Allen et al., 2019). Robust screening methods and relevant translatable disease models are being used for the development of new therapies and preclinical testing (Wirtz et al., 2017; Patil et al., 2019). State-of-the-art molecular imaging of inflammation, such as Single Photon Emission Computed Tomography (SPECT), Positron Emission Tomography (PET), or Magnetic resonance imaging (MRI) is being used to improve our understanding of the pathophysiology of diseases. Longitudinal *in vivo* bioluminescence and fluorescence imaging has become widely used tool for studying the inflammation process in laboratory animals (Mezzanotte et al., 2017; Schmidtchen and Puthia, 2020).

This special Research Topic ‘Methods and Applications in Inflammation Pharmacology: 2022 focuses on pharmacological interventions and state-of-the-art methods during inflammatory conditions. A total of five original articles and one review were published.

Dandelions (*Taraxacum* spp.) have been used as a medicinal herb for a long time. Dandelions contain various bioactive substances which are reported to have pharmacological properties. Li et al. investigated the anti-inflammatory effects of Dandelion extract in LPS-induced RAW264.7 macrophages and copper sulfate (CuSO4)-induced zebrafish larvae. The dandelion extract reduced the LPS-induced inflammatory response in RAW264.7 cells by regulating polarization and apoptosis. Showing a therapeutic potential, the dandelion extract also reduced the CuSO_4_-induced inflammatory response in zebrafish larvae (Li et al.).

Icariin, a flavonoid compound isolated from plants of the *Epimedium genus*, has shown anti-inflammatory, immunoregulatory, and antibacterial properties. Li et al. investigated the effects of icariin on inflammation-associated intestinal barrier function impairment and showed that it attenuated the expressions of Occludin, Claudin1, and Claudin5 in rat colon (Li et al.). Icariin alleviated TNF-α-induced Occludin disruption and epithelial barrier impairment by decreasing miR-122a expression in Caco-2 cell monolayers. In another study, Shao et al. demonstrated that icariin protects against cartilage endplate degeneration and calcification under intervertebral disc degeneration conditions, and the associated mechanism may be related to Nrf-2/HO-1 mediated mitophagy activation and ferroptosis inhibition (Shao et al.). Both of these studies show the therapeutic potential of icariin during inflammatory disorders.

Chen G. et al. Investigated the therapeutic potential of active component formulation (ACF) from Huanglian Jiedu Decoction during LPS-induced systemic inflammation. ACF components showed a good binding ability to MD-2 and ACF treatment reduced inflammatory cell infiltration and organ damage in rat LPS-induced sepsis model (Chen et al.).

In another study, Ahsan et al. showed that *Saussuria lappa* extract has immunomodulatory effects and reduced the number of colonizing bacteria in the liver, spleen, and lungs and also lowered the levels of neutrophils and interleukin eight in *Acinetobacter baumannii* mouse infection model (Ahsan et al.). Finally, Xiao et al. reviewed the role of inhibitor of nuclear factor kappa-B kinase ε (IKKε) in metabolic diseases and summarized the structural characterization, physiological function, and pathological role of IKKε in metabolic diseases, and discussed small molecule inhibitors of IKKε (Xiao et al.).
